# P-1956. Hospitalizations for Coccidioidal Meningitis: A Review of Outcomes and Trends in an Endemic Setting from 2020-2024

**DOI:** 10.1093/ofid/ofaf695.2124

**Published:** 2026-01-11

**Authors:** Michelle Fang, Jigar Patel, Bianca Torres, Jenessa Olson, Carlos M D’Assumpcao, Rasha Kuran, Royce H Johnson, Shikha Mishra

**Affiliations:** Kern Medical, Bakersfield, CA; Kern Medical, Bakersfield, CA; Valley Fever Institute at Kern Medical, Bakersfield, California; Western University, Pomona, California; Kern Medical, Bakersfield, CA, Bakersfield, California; Kern Medical Center, Bakersfield, California; Kern Medical Center, Bakersfield, California; Kern Medical-UCLA, Bakersfield, California

## Abstract

**Background:**

Coccidioidal meningitis (CM) is a form of disseminated coccidioidomycosis associated with significant morbidity and mortality. Based on clinician observations of increased numbers and complexity of CM hospitalizations at Kern Medical (KM), a community teaching hospital in Bakersfield, CA, we sought to characterize recent hospitalizations at KM for CM.

**Figure 1.**
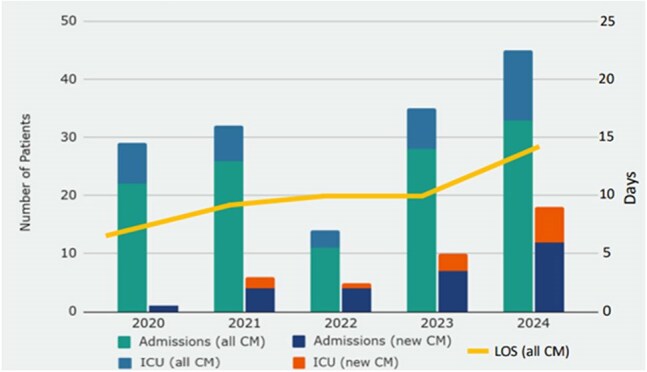
KM Hospitalizations for CM from 2020-2024

**Methods:**

This Institutional Review Board-approved retrospective review included hospitalizations for CM at KM from January 1, 2020 to December 31, 2024. Hospital admissions with an International Classification of Diseases, tenth revision (ICD-10) code for CM (B38.4) were extracted from the electronic medical record, then manually reviewed to exclude admissions unrelated to CM management and gather additional demographics and outcomes data. Characteristics of interest for both the overall population and the subgroup of hospitalizations for a new CM diagnosis included length of stay (LOS), intensive care unit (ICU) admission, disposition, and trends over time.

**Results:**

Across the 5-year time frame, 203 admissions (99 unique patients) were identified with ICD-10 code B38.4. Of these, 120 admissions (73 unique patients) were determined to be primarily for management of CM, with 28 admissions for new CM diagnoses. Mean hospitalizations per patient was 1.6 (range 1-6 admissions). Mean age at time of admission was 48 years, with men accounting for 84% of hospitalizations. Mean LOS was 10 days (range 1-80 days) with 5% in-hospital mortality. Admission to the ICU was required during 29% of encounters, including 12% with endotracheal intubations. Of the hospitalizations for a new CM diagnosis, mean LOS was 15 days (range 2-66 days) with 14% in-hospital mortality. ICU admission was required in 43% of new CM hospitalizations, with 25% requiring intubation. Trends in CM admissions over time are described in Figure 1.

**Conclusion:**

CM is a devastating manifestation of coccidioidomycosis and this review highlights the burden of CM on clinical outcomes and healthcare resource utilization in an endemic setting. The apparent increase in hospitalizations for both new diagnoses and exacerbations of CM over a 5-year period is alarming and calls for further investigation to validate this trend and identify potential contributing factors.

**Disclosures:**

All Authors: No reported disclosures

